# Incidence and risk factors of herpes zoster among adult renal transplant recipients receiving universal antiviral prophylaxis

**DOI:** 10.1186/s12879-015-1038-1

**Published:** 2015-07-24

**Authors:** Ioanna D. Pavlopoulou, Stavroula Poulopoulou, Christina Melexopoulou, Ioanna Papazaharia, George Zavos, Ioannis N. Boletis

**Affiliations:** Paediatric Research Laboratory, National and Kapodistrian University of Athens, Faculty of Nursing, Athens, Greece; Athens University of Economics and Business, Athens, Greece; Renal Transplant Unit, “Laiko” General Hospital, National and Kapodistrian University, Faculty of Medicine, Athens, Greece; Postgraduate Program, National and Kapodistrian University of Athens, Faculty of Nursing, Athens, Greece

**Keywords:** Herpes zoster, HZ, Incidence, Complications, Post- herpetic neuralgia, PHN, Risk factors, Renal transplantation, Adults

## Abstract

**Background:**

Herpes zoster (HZ) is a significant cause of morbidity and complications in adult renal transplant recipients. We determined the incidence, complications and risk factors for the development of HZ after renal transplantation in a setting using universal antiviral prophylaxis.

**Methods:**

The medical files of all adult renal transplants, performed between 2004 and 2008, were retrospectively reviewed to assess the clinical characteristics and risk factors of HZ. Incident cases of HZ were determined and the probability of developing post-transplant HZ for all subjects was calculated using the Kaplan Meier method. A multivariable Cox proportional hazards model was applied to assess the risk factors associated with the development of HZ.

**Results:**

A total of 450 patients were eligible with a median follow up of 38 months. Twenty nine subjects (6.4 %) developed HZ, the median time to onset was 18 months, only three of them (10.3 %) required hospitalization, and none developed disseminated or visceral disease and death directly attributed to zoster. However, high rates of post-herpetic neuralgia (48.7 %) were observed. Overall incidence was calculated at 20.6 cases per 1000 patient-years of follow-up. Following multivariate analysis, increased age ≥ 60 years old, positive pre-transplant history of varicella related disease and administration of rejection treatment conferred an increased risk of 4.00-fold (CI: 1.79- 8.92), 16.00-fold (CI: 4.62- 55.52), and 5.57-fold (CI: 1.56- 19.84) respectively, for the development of post-transplant zoster.

**Conclusions:**

HZ remains a common complication after renal transplantation in adults under current immunosuppession protocols and universal antiviral prophylaxis.

**Electronic supplementary material:**

The online version of this article (doi:10.1186/s12879-015-1038-1) contains supplementary material, which is available to authorized users.

## Background

Herpes zoster (HZ) represents the reactivation of the varicella zoster virus (VZV), which establishes lifelong latency in cranial- nerve or dorsal-root ganglia after the primary infection that had caused chickenpox [1, 2]. The risk of HZ increases with impaired specific cell mediated immunity mainly as a result of advancing age [3–6] or acquired immunosuppression [7–11]. It has been estimated that the incidence of HZ in solid organ transplant (SOT) patients is 10- to 100-fold higher than in the general population, ranging between 18.3 to 55.1 cases per 1000 person-years, with lung and heart recipients appearing to be at highest risk [12, 13]. The most common manifestation of VZV reactivation in SOT is cutaneous HZ, involving ≤ 2 adjacent dermatomes, however, atypical clinical findings, disseminated disease, visceral involvement and lethal outcome have been described [14]. In addition, 20-40 % of the transplant recipients will develop post-herpetic neuralgia (PHN) as a secondary complication, significantly greater than the rate in immunocompetent populations [13].

Risk factors for the development of HZ in SOT have not been well defined due to the lack of large prospective trials. Longitudinal studies have demonstrated that older age, race and type of transplant may increase the rates of HZ in solid organ recipients [12, 15]. The development of novel immunosuppressive agents has dramatically improved the survival of all SOT recipients, including renal transplant patients, however, there is accumulating evidence that the use of the above agents may more frequently induce certain viral infections, including VZV [16–20]. Although existing studies have not been designed or powered to determine the effect of specific immunosuppressive agents or respective combinations on VZV incidence, mycophenolate mofetil (MMF) has been suggested as potential risk factor [20].

According to updated clinical practice guidelines, the live attenuated herpes zoster vaccine (Zostavax®, Merck & Co., Inc., USA) is recommended to pre- transplant candidates aged ≥ 60 years and varicella-positive candidates aged 50-59 years who are not severely immunocompromised if transplantation is not expected within 4 weeks [21].

The aim of the present study was to evaluate the incidence, complications and risk factors for the development of HZ in a large, single- center renal transplant cohort using current immunosuppressant and antiviral prophylaxis protocols.

## Methods

### Patient population and study design

We reviewed the records of all adult patients who underwent renal transplantation (RT) between January 1^st^, 2004 and December 31^st^, 2008 at the Renal Transplant Unit of “Laiko” General hospital, in Athens, Greece. This is a tertiary hospital, where more than 40 % of the country’s kidney transplantations are performed. All patients originate from Central and Southern Greece and remain under regular follow up by the above transplant unit. Demographic, clinical and laboratory information were abstracted from the patients’ medical records and included the following: Age, gender, time of transplant, type of donor, immunosuppressive protocol, episodes of acutegraft rejection, history of VZV related disease, pre-transplant VZV serologic status, antiviral prophylaxis, distribution of HZ and related complications, including hospital admission. The diagnosis of HZ was clinical and was based on the characteristic signs and symptoms, including the classic appearance and distribution of the rash. Post-herpetic neuralgia was defined as pain persisting in the affected area for more than 30 days after the development of the rash. The date of last data collection was February 28^th^, 2009 which allowed for a minimum follow up of two months for each patient. Individuals with early loss of graft and follow-up or survival of less than 2 months after RT were excluded from the analysis. In order to clarify further their pre- transplantation history of varicella or HZ, all patients were contacted by telephone. At this point, they were informed about the aim of the present study and a verbal consent was obtained. The study protocol was approved by the institution review board of “Laiko” General Hospital.

### Immunosuppressive regimen

The immunosuppressive regimens for induction therapy after RT included anti-interleukin (IL)-2 receptor antibody (basiliximab or daclizumab) or anti-thymocyte globulin (ATG). Triple combinations of various immunosuppressive agents including tacrolimus (TAC) or cyclosporine (CsA), mycophenolate mofetil or mycophenolate sodium (MPA), everolimus or rapamune (mTORi) and methylprednizolone (MD), were used as maintenance therapy.

### Herpesvirus prophylaxis

Antiviral prophylaxis was prescribed to all RT recipients according to donor and recipient cytomegalovirus (CMV) status. All CMV- IgG positive recipients were treated with a seven day course of intravenous ganciclovir, at a dose of 125 mg/kg, followed by three months of oral valganciclovir, with dose adjustments according to the patient’s creatinine clearance. Patients who were CMV- IgG negative and received an allograft from a CMV- IgG positive donor were treated with the same protocol for six months. Finally, recipients who were CMV-IgG negative and their donor was also CMV-IgG negative did not receive any prophylaxis.

### Statistical analysis

Statistical package for the social sciences (SPSS) version 18 was used to conduct the statistical analysis and p- value <0.05 was considered statistically significant. Continuous and categorical variables were summarized as appropriate. Differences of the continuous variables between patients who developed HZ and those who did not, were analyzed using Mann–Whitney tests when the assumptions required by the parametric counterpart tests were not met andthe primary endpoint was the incident of HZ. The incident rates of HZ were estimated by the number of HZ’s incidents developed in our cohort, divided by the sum of the person-time of the transplanted population. The probability of developing post-transplant HZ for all subjects and between subgroups of interest was calculated using the Kaplan Meier method. Log-rank tests and Walt test (Cox model) were used for comparison of the probability of developing post-transplant HZ between groups of interest. To determine risk factors for the development of HZ following KT, univariate and multivariable Cox proportional hazards model were used. Cox proportional hazards regression was used to model the association of the incident of HZ to patients and clinical characteristics and to estimate hazard ratios (HRs) along with their 95 % confidence intervals (CIs). In multivariate models, with the use of the forward selection method, with removal criterion of 10 %, the association to outcome of each of these factors in the presence of the others in the model was explored. For analyses, available predictor variables included: age, gender, pre-transplant history of VZV, induction agent, rejection treatment and immunosuppressive regimen. Immunosuppressive regimen was analyzed as a time-dependent variable in the Cox regression model. Post-transplant zoster development was also used as a time-dependent effect in mortality and graft failure Cox models.

## Results

### Characteristics of kidney transplant recipients

A total of 479 adults underwent RT over the study period. Τwenty-eight were excluded from the analysis because of lack of follow up for at least 60 days post- transplantation. One additional patient was excluded because he received both kidney and liver transplantation. Finally, 450 patients with a mean age of 45 ± 13 (range 18-73) years at transplantation were eligible for further analysis. The characteristics of these patients are presented in Table 1. Eleven deaths (2.44 %) occurred during the study period, eight of those (72.8 %) with functioning graft. As illustrated, a great proportion of the study population (44.7 %) lacked information regarding history of varicella or HZ before transplantation, collected either from medical records or by telephone interview. Moreover, pre- transplant VZV serology was not available for any of the participants, as this was not a standard requirement in our department at the time of the present study. According to clinical data available for 252 patients, the majority (79.7 %) reported a pre-transplant varicella related disease. Of these, 180 patients reported a positive history of chickenpox, while the remaining twenty- one suffered an episode of pre-transplant zoster. Two of the latter presented a recurrence of HZ post- transplantation. No patient was vaccinated against varicella before transplantation, as the respective vaccine was only licensed in 2008 in our country.The participants’ immunosuppressive regimens were recorded at 3, 6, 12 and 18 months post- transplantation and are presented in Table 2.  As shown, mycophenolate mophetil or mycophenolate sodium were included in the great majority of the patients’ immunosuppressive regimens, at all time points.

**Table 1:** Clinical characteristics of kidney transplant patients, (*n* = 450)

Characteristics	N (%)
*Age at transplantation (years)*	45 ± 13
<60	377 (83.8)
≥ 60	73 (16.2)
*Gender*	
Male	293 (65.1)
Female	157 (34.9
*Follow up time (months); median (range)*	38 (2-71)
*Type of donor*	
Live related donor	192 (42.7)
Diseased donor	258 (57.3)
*Number of transplant*	
First	436 (96.9)
Second	11 (2.4)
Third	3 (0.7)
*Induction agent*	
Daclizumab	237 (52.7)
Basiliximab	176 (39.1)
Anti-thymocyte globulin	23 (5.1)
None	14 (3.1)
*Death at last follow-up*	
No	439 (97.6)
Yes	11 (2.4)
*Graft functionality at death*	
Unknown	1 (9.1)
Graft in function	8 (72.7)
Graft not in function	2 (18.2)
*Rejection*	
No	417 (92.6)
Yes	33 (7.4)
*Pre-transplant VZV disease*	
No	51 (11.3)
Yes	201 (44.7)
Unknown	198 (44)

**Table 2:** Immunosuppressive regimen according to follow-up time in kidney transplant patients, (*n* = 450)

	*3 months N (%)*	*6 months N (%)*	*12 months N (%)*	*18 months N (%)*
TAC/MPA/MP	212 (47.1)	217 (48.2)	215 (47.8)	210 (46.7)
CsA/MPA/MP	145 (32.2)	144 (32.0)	131 (29.1)	114 (25.3)
TAC/mTORi/MP	72 (16)	60 (13.3)	53 (11.8)	50 (11.1)
CsA/mTORi/MP	1 (0.2)	1 (0.2)	1 (0,2)	0 (0)
MPA/mTORi/MP	16 (3.6)	15 (3.3)	24 (5,3)	24 (5,3)
Double immunosuppressive regimen	2 (0.4)	3 (0.7)	15 (3,3)	36 (8.0)
None/unknown	2 (0.4)	10 (2.2)	11 (2.4)	16 (3.6)

TAC: tacrolimus; MPA: mycophenolatemofetil or mycophenolate sodium; MP: methylprednisolone; CsA: cyclosporine; mTORi: rapammune or everolimus

### Herpes zoster events

Twenty- nine out of 450 eligible kidney recipients (6.4 %) developed HZ during the study period, and the mean time to onset was 22.21 ± 14.49 months (median 18 months, range 5-58). The mean age of the patients who manifested zoster was higher when compared to the age of those who did not (median age 50 versus 45 years old; Mann–Whitney test p-value = 0.006). The clinical characteristics of these patients are illustrated in Table 3. All episodes involved a single dermatome, there were no recurrences nor disseminated disease and no patient sustained primary varicella infection. As shown, the majority (89 %) received oral antiviral treatment and were managed as outpatients, two subjects had a positive history of pre- transplantation HZ and 48 % suffered PHN. None of the 21 CMV-IgG negative recipients who received graft by a CMV-IgG negative donor, developed zoster.

**Table 3:** Characteristics of herpes zoster in kidney transplant patients (*n* = 29)

Characteristics	N (%)
*Time to onset (months): median (range)*	18 (4 - 58)
*Age (years): mean ± SD*	52 ± 12
*Distribution*	
Lumbosacral	6 (20.7)
Thoracic or abdominal	9 (31)
Facial	5 (17.2)
Extremities	4 (13.8)
Visceral	0 (0)
Unknown	5 (17.2)
*Hospital admission*	
No	26 (89.7)
Yes	3 (10.3)
*Antiviral treatment*	
Valacyclovir	11 (37.9)
Acyclovir (i.v)	2 (6.9)
Acyclovir (p.o)	12(41.4)
Famciclovir	2 (6.9)
Unknown	2 (6.9)
*Pre-transplant VZV disease*	
No	2 (6.9)
Yes	27 (93.1)
*PHN*	
No	15 (51.7)
Yes	14 (48.3)

SD: standard deviation; VZV: varicella zoster virus; PHN: post- herpetic neuralgia

**Fig. 1:**
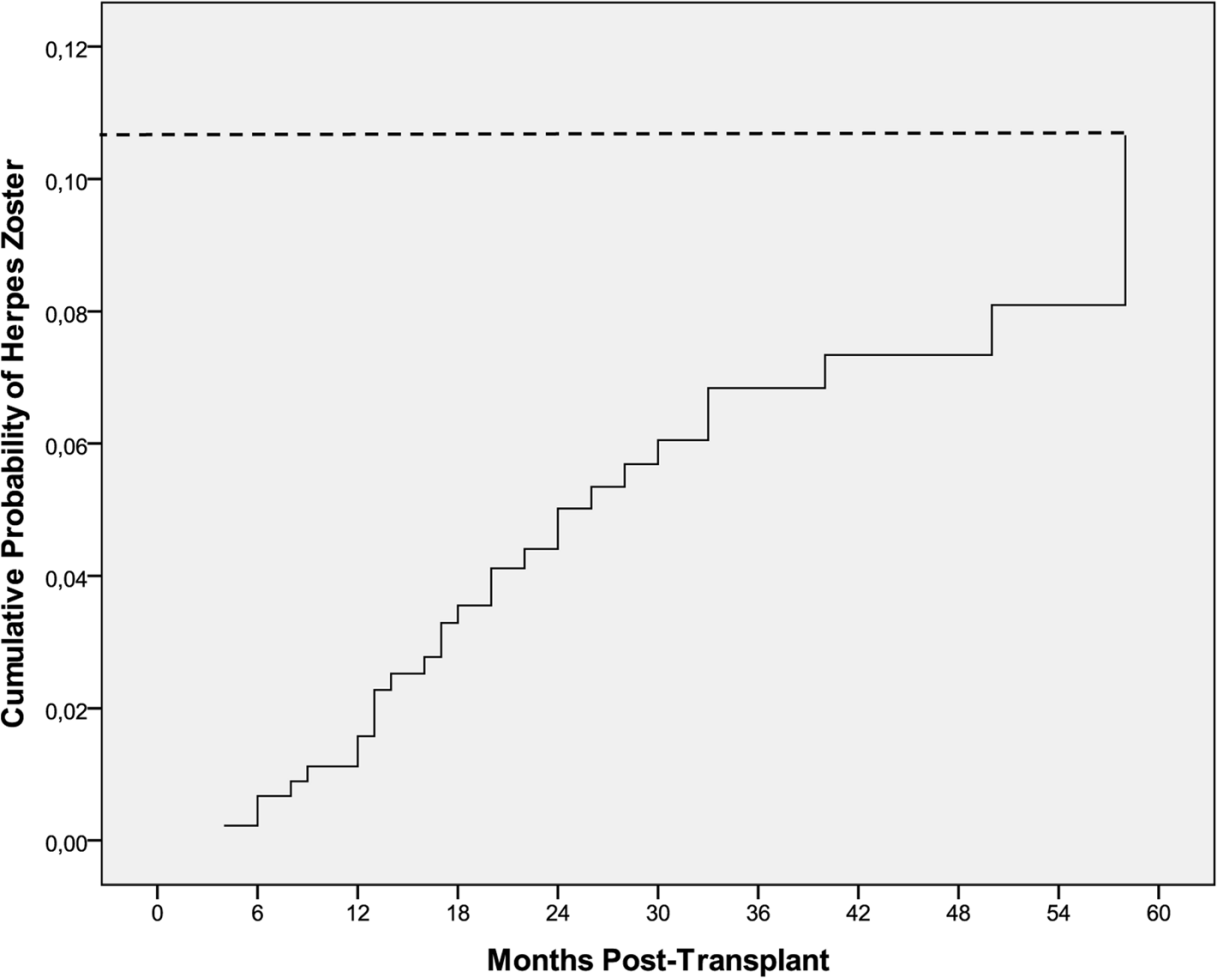
Cumulative probability of herpes zoster after kidney transplantation

The cumulative probability of HZ using the Kaplan-Meier method was 0.7 % at 6 months, 1.6 % at 12 months, 5 % at 24 months, 6.8 % at 36 months and 10.7 % at 60 months following transplantation (Fig. 1). The estimated total incidence rate of HZ was 20.6 cases per 1000 person- years based on the exact time each person contributed on the study.

Furthermore, we analyzed the cumulative incidence of HZ in two separate age groups; those younger than 60 versus those equal or older than 60 years at time of transplant. In the younger age group, the cumulative incidence of HZ, using the Kaplan-Meier method, was 0.3 % at 6 months, 1.1 % at 12 months, 4.5 % a t 24 months, 6.2 % at 36 months and 6.8 % at 60 months. In the older group, the respective incidence increased from 2.9 % at 6 months, to 4.3 % at 12 months, 7.7 % at 24 months, 10.1 % at 36 months and 56.4 % at 60 months following transplantation (log-rank p-value = 0.014, see Additional file 1: Figure S1). The overall incidence of HZ was higher for patients ≥60 years old throughout the entire follow-up period. In particular, the estimated average incidence rate of HZ in patients < 60 years of age was 16.7 cases per 1000 person- years, while in patients ≥ 60 years, 43 cases per 1000 person- years, respectively.

### Herpes zoster risk factor analysis

Univariate analysis was performed to determine whether age at transplantation, gender, history of pre-transplant VZV related disease, induction agent and rejection treatment were risks for the development of HZ (Table 4.). By modelling age as categorical variable, patients ≥ 60 years old conferred a 2.598-fold increased risk for HZ development when compared to those aged < 60 years (CI 1.180 - 5.721; p-value = 0.018). In contrast, no association of HZ and gender was detected, however, we observed a trend of increased probability for zoster in women after the first year post-transplant (see Additional file 1: Figure S2). Patients with a pre- transplant history of VZV related disease were 6.22-fold more likely to develop HZ when compared to those with negative history (CI 0.844- 45.847; p-value =0.07, Table 4). As shown in Additional file 1: Figure S3, the risk increased with time post-transplantation. No significant association between induction agent and risk for HZ was observed. Finally, no interaction was noted between the different immunosuppressive regimens or between rejection treatment and the risk for post- transplant HZ, when analyzed by use of time dependent models. However, in patients who received rejection treatment the risk for HZ was slightly increased.

**Table 4:** Univariate and multivariate Cox analysis of risk factors for the development of herpes zoster in kidney transplant patients, (*n* = 450)

Risk Factors	Hazard ratio	95 % Confidence Interval	p-value
Univariate analysis			
*Age, (years)*			
< 60	1.00 (referent)		
≥ 60	2.598	1.180 - 5.721	0.018
*Gender*			
Female	1.00 (referent)	-	-
Male	0.671	0.323-1.396	0.286
*Pre-transplant VZV disease*			
No/Unknown	1.00 (referent)	-	-
Yes	10.777	3.262-35.612	0.0001
*Induction agent*			
None	1.00 (referent)	-	-
Anti-thymocyte globulin (ATG)	0	0- n.e^a^	0.977
Basiliximab	0.36	0.81-1.612	0.182
Daclizumab	0.39	0.89-1.709	0.211
*Rejection treatment*			
No	1.00 (referent)	-	-
Yes	2.08	0.624-6.930	0.233
Multivariate analysis			
*Age, (years)*			
< 60	1.00 (referent)		
≥ 60	3.995	1.790-8.915	0.001
*Pre-transplant VZV disease*			
No/Unknown	1.00 (referent)	-	-
Yes	16.022	4.624-55.522	0.008
*Rejection treatment*			
No	1.00 (referent)	-	-
Yes	5.566	1.562-19.835	0.0001

^a^n.e.: not estimated

Following multivariate analysis, age group (<60 or ≥ 60 years) at time of transplant and positive pre-transplant history of VZV remained statistically significant risk factors for HZ. In particular, individuals ≥ 60 years old presented an increased by 3.0 % risk, while those with positive pre-transplant history of VZV an increased by 15.0 % probability of shingles when compared to the individuals with a respective negative history, following transplantation. Moreover, patients who received anti-rejection treatment were 5.6 times more likelyto develop HZ when compared to those who did not receive such treatment (Table 4).

When the development of HZ was analyzed as time-dependent covariate, a statistically significant increased mortality was found in patients who developed HZ compared to those that did not. In specific, patients who developed HZ had an 8.076-fold increased mortality versus those who did not (CI: 2.079-31.376; p-value = 0.003, Finally, there was no evidence of increased graft failure among subjects manifesting post-transplant zoster.

## Discussion

The present study provides information about the epidemiology of herpes zoster among adult renal transplant patients in the country’s largest transplant center, over a five- year period. This transplant unit uses a consistent immunosuppression protocol along with universal prophylaxis against cytomegalovirus, while all renal recipients receive long term follow up by the same medical team. Our results revealed an overall incidence rate of HZ of 20.6 cases per 1000 person-years, while age ≥ 60 years at time of transplant, positive pre-transplant history of varicella- zoster related disease and administration of anti- rejection treatment were identified as significant risk factors for the development of shingles; most HZ cases were managed as outpatients, there was no disseminated disease, however, a high proportion of individuals suffered post-herpetic neuralgia.

The incidence of post-transplant HZ seems to differ according to transplant organ probably reflecting the various levels of induced immunosuppression. Consequently, a higher incidence of HZ has been observed among heart transplants, followed by kidney and finally, liver transplants who generally require less immunosuppression [22]. Estimates for overall zoster incidence in renal transplants have ranged between 24.7 and 44.6 per 1000 person-years in previous retrospective studies, but these variations mostly indicate the intensity of immunosuppression as well as the differences between the prophylactic antiviral protocols [12, 15, 20, 23]. The respective incidence rate among our study population, albeit lower, is comparable to Arness et al. [15], who have also used induction therapy and antiviral prophylaxis against CMV in the great majority of their patients. Despite the retrospective data collection in the present study, it is unlikely that the true incidence of zoster is underestimated, since all patients had continuing follow-up by the same team of doctors.

The time to zoster development in SOT has been previously reported between 6.6 months to 4.9 years, while in our group of patients the respective median onset time was 18 months [15, 19, 23, 24]. It is evident that these variations probably reflect differences in follow-up time and therefore, prolonged immunosuppression. As shown before [15], when we used the Kaplan-Meier method to evaluate the probability of developing HZ in the post transplant period, we found that this probability, although very low (0.7 %) in the first six months, increased rapidly over time, reaching 10.7 % after five years.

The majority of the 29 individuals who developed zoster following transplantation experienced mild disease, involving a single dermatome and were managed as outpatients. Only three patients required hospitalization because of intense pain, vomiting and dehydration. However, we report a high rate of PHN (48.3 %) contrary to other authors who have reported PHN between 5-42 % of solid organ recipients [15, 19]. Risk factors for postherpetic neuralgia include older age and greater severity of the prodrome, rash, and pain during the acute phase [25]. Our findings cannot be attributed to the difference in the age distribution between the group of patients who developed PHN and those who did not, since the mean age between the two groups was similar. It is noteworthy, however, that all three subjects needing hospitalization, experienced PHN.

Interestingly, we observed an 8.076-fold increased mortality risk in patients who developed zoster compared to those who did not, and this was not directly related to viral infection mortality. To the best of our knowledge, this has not been reported before, however, this should be interpreted cautiously since it was only based on few events and was not addressed in the multivariate analysis. A possible explanation could be that post-transplant VZV reactivation is an independent indicator of over-immunosuppression per se, thus predisposing to higher mortality; this is also consistent with the fact that we did not have any evidence of increased graft failure among the individuals with zoster.

Age has been a traditional risk factor for zoster among immunocompetent as well as among adults with immunosuppression [12, 15, 26, 27]. Our data confirmed that age equal or greater than 60 years at transplantation, conferred an increased by 3 % risk for VZV reactivation when compared to the younger age group.

Women, especially those belonging to older age groups, have been described to be at increased risk for zoster in the general population [28]. It has not been clarified though, whether female gender represents a true biological mechanism of susceptibility or this may be attributed to increased disease documentation, since women with zoster appear to seek medical advice more often than men [29]. We only identified an increased trend of zoster among our female transplant recipients after the first year post- transplant.

We demonstrated that pre-transplant VZV related disease was a statistically significant risk factor for shingles following transplantation. As the large majority of adults who undergo transplantation have likely experienced varicella during childhood, it would possibly be more appropriate to link post- transplant HZ to zoster events before transplantation. However, there is uncertainty about the degree and duration of protection from previous HZ in healthy persons, and even more so among those with immunosuppression, since the threshold under which HZ occurs is unknown [30–32]. Recent advances in the understanding of the pathogenesis of HZ indicate that, besides cell-mediated immunity dysfunction, additional factors including co-infection with different strains of VZV and genetic susceptibility may have a role in the reactivation of the latent virus [30]. Since the information of pre-transplant VZV related disease was mainly self reported and there were only few individuals with zoster events prior to transplant, we chose to link the risk of VZV related disease as a whole, in our cohort. As shown recently, 92.5 % of unvaccinated Greek adults of younger age, presented serologic evidence of immunity against chickenpox. In particular, seropositivity was identified in 100 % of those with positive, but also in 53 % and 91 % of those reporting a negative or unknown history of disease, respectively [33]. More than half of our patients reported a negative or unknown history of varicella or zoster, therefore, in accordance with the above findings, we can assume that the correlation of positive pre-transplant history of VZV related disease with post-transplant zoster is underestimated in our analysis.

Intensive immunosuppression including induction or acute anti- rejection treatment has been associated with increased risk of zoster [19, 22]. In particular, the use of MPA has been described as a risk factor for zoster by some investigators [15]. However, this has not been a consistent finding, as the scientific evidence concerning the risk of specific immunosuppressive agents or combinations for the development of HZ in SOT remains insufficient. As shown, no such association was found in our study population, and this could be attributed to the fact that the great majority of our patients were treated with one of the MPA’s during the whole study period as was also the case with induction treatment. In contrast, anti-rejection therapy conferred a significantly increased risk for zoster in the post-transplant period, and this, despite the fact that the incidence of acute rejection among our study participants was extremely low (2.7 %).

According to previous investigators, pre-emptive rather than universal prophylaxis against CMV increases the risk of zoster following SOT [20, 34]. As our patients were on prophylactic antiviral treatment and no one received pre-emptive, we were not able to demonstrate any similar effect. Nevertheless, one could speculate that universal prophylaxis may have contributed to the delayed onset of zoster among our population.

The epidemiology of HZ among both healthy and immunocompromised individuals will remain an ongoing challenge in the advent of the aging population, the use of novel immunosuppressive agents and the implementation of universal vaccination against primary varicella in childhood. According to recent data, the incidence of HZ among the general population has decreased [35] following prophylactic varicella vaccination in childhood, however, other investigators report an increased herpes zoster incidence in a setting of increased varicella vaccine coverage [36]. It is evident that continuous surveillance and research are needed to demonstrate how the lack of natural boosting will affect SOT patients. In Greece, varicella vaccination has been introduced in the National Immunization Program since 2008 while the live attenuated vaccine against herpes zoster (Zostavax®, Merck & Co., Inc., USA) was licensed in October 2014, awaiting official recommendations. The latter, has been shown to be safe and protective in the immunocompetent elderly population [37]. Despite recent guidelines proposing the use of Zostavax among certain groups of immunocompromised adults, there has been no study evaluating whether pre-transplant immunization against HZ would decrease the risk of post-transplant zoster in SOT [21, 31, 38]. Herein, additional issues to be addressed include limited vaccine efficacy in patients with end stage renal disease awaiting transplantation as well as optimal time interval between vaccine administration and transplantation in terms of safety. An investigational adjuvanted subunit vaccine (called HZ/su, GlaxoSmithKline Biologicals) has proven safe and effective among older healthy adults and if studied in the SOT population, it might prove a useful alternative to the live attenuated vaccine because of the reduced risk of disease resulting from replication [39].

### Study limitations and strengths

This study has a number of limitations. It was a retrospective, single center study and can, therefore, not be generalized. In addition, as pre-transplant VZV serology was not available for any of our patients, self reported VZV related disease, being also subject to recall bias, underestimates the proportion of subjects with primary immunity against VZV. Finally, it is evident that in a larger study many of the trends that appeared when evaluating risk factors might be different. Despite the above limitations, this is the first study to provide data about the epidemiology, complications and risk factors for the development of HZ in renal transplant recipients in our country.

## Conclusions

In the present study we have shown that HZ is a frequent cause of morbidity among adult renal transplant recipients in the country’s largest transplant center, that uses a consistent immunosuppression and a universal antiviral prophylaxis protocol. Among our transplant patients with zoster, we observed high rates of PHN and a higher risk of mortality that was not directly related to acute VZV disease. The latter should be interpreted with caution as this was not addressed in multivariate analysis. Older age, history of pre-transplant VZV related disease and use of anti- rejection treatment were identified as independent risk factors for shingles following transplantation. In view of the recent licensure of the live attenuated vaccine Zostavax in our country, along with the updated recommendations for vaccination of the immunocompromised, the above results may provide useful evidence in order to design optimal strategies to prevent HZ among individuals awaiting renal transplantation.

## Additional file

Additional file 1: Figure S1. Cumulative probability of post-transplant herpes zoster among patients ≥ 60 versus < 60 years of age; **Figure S2.** Cumulative probability of post-transplant herpes zoster according to gender; **Figure S3**. Cumulative probability of post-transplant herpes zoster among patients with pre- transplant history of HZ. (DOC 107 kb)
