# Supplementary material for: Incidence and risk factors of herpes zoster among adult renal transplant recipients receiving universal antiviral prophylaxis
Source: BMC Infect Dis. 2015 Jul 24;15:285. doi: 10.1186/s12879-015-1038-1 (PMC4513398; doi:10.1186/s12879-015-1038-1)

**Figure S1** Cumulative probability of post-transplant herpes zoster among patients ≥ 60 versus < 60 years of age

**Figure S2** Cumulative probability of post-transplant herpes zoster according to gender


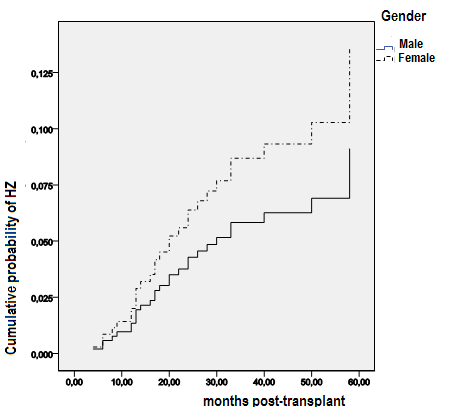


**Figure S3** Cumulative probability of post-transplant herpes zoster among patients with pre- transplant history of HZ.


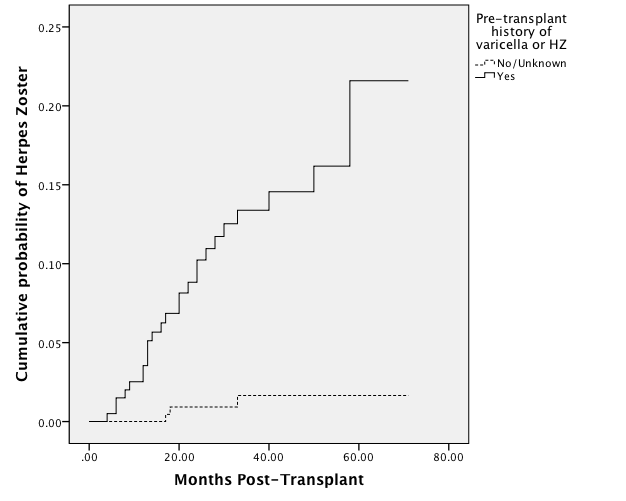

Supplement: Additional file 1: Figure S1. — Cumulative probability of post-transplant herpes zoster among patients ≥ 60 versus < 60 years of age; Figure S2. Cumulative probability of post-transplant herpes zoster according to gender; Figure S3. Cumulative probability of post-transplant herpes zoster among patients with pre- transplant history of HZ. (DOC 107 kb) [file 12879_2015_1038_MOESM1_ESM.doc]
